# Effects of Remote Ischemic Conditioning on Cardiovascular Responsiveness in Healthy Individuals

**DOI:** 10.3390/life15060842

**Published:** 2025-05-23

**Authors:** Inga Kiudulaite, Jelena Cesarskaja, Mante Eidininkiene, Zivile Pranskuniene, Andrius Pranskunas

**Affiliations:** 1 Department of Intensive Care Medicine, Lithuanian University of Health Sciences, Eiveniu g.2, 50161 Kaunas, Lithuania; inga.kiudulaite@lsmu.lt (I.K.); jelena.cesarskaja@gmail.com (J.C.); mante.bruzaite@gmail.com (M.E.); 2 Institute of Pharmaceutical Technologies, Lithuanian University of Health Sciences, Sukileliu pr. 13, 50166 Kaunas, Lithuania; zivile.pranskuniene@lsmu.lt; 3 Department of Drug Technology and Social Pharmacy, Lithuanian University of Health Sciences, Sukileliu pr. 13, 50166 Kaunas, Lithuania

**Keywords:** remote ischemic conditioning, passive leg raising, systemic hemodynamics, peripheral perfusion, cardiovascular response

## Abstract

The remote ischemic conditioning (RIC)-induced changes in systemic hemodynamics or circulatory reactivity are unclear. Therefore, we aimed to evaluate the effect of a single bout of RIC on the passive leg raising (PLR)-induced cardiovascular response. This prospective study included 36 healthy volunteers (median age: 24 years). Systemic hemodynamic indices were assessed through the following sequential steps: with the participant in a supine position, during the first PLR maneuver, before RIC, after RIC, during the second PLR maneuver, and with the participant in a supine position. The perfusion index (PI) was measured during PLR before and after RIC. We found no significant differences before and after RIC in the proportion of responders during PLR (participants with stroke volume (SV) change ≥ 10%, 61% vs. 47%, *p* = 0.180). There was a strong correlation between SV changes during the two PLR tests (r_s_ = 0.80, *p* < 0.001). PLR significantly increased the PI before and after RIC. However, there was no significant difference before and after RIC in the PLR-induced PI changes (*p* = 0.944). Our findings suggest that a single bout of RIC has no effect on PLR-induced cardiovascular responses in terms of changes in systemic hemodynamic and peripheral perfusion indices.

## 1. Introduction

Remote ischemic conditioning (RIC) is a therapeutic approach that involves exposing tissues to brief, repeated periods of restricted blood flow (ischemia) followed by the restoration of blood flow (reperfusion). This process helps reduce ischemia–reperfusion injury in distant organs or tissues that are not directly subjected to the initial ischemic event [1,2]. The most widely used RIC technique consists of three to four cycles of inflating a standard blood pressure cuff on the upper arm or thigh for 5 min, followed by a 5 min deflation period [1]. Although the signaling pathways are not completely understood, the RIC stimulus is believed to be transmitted through humoral, neural, and systemic pathways [3]. Human studies have indicated that a single bout of RIC improves the endothelium-dependent vasodilation of remote conduit arteries [4,5]. RIC may promote nitric oxide (NO) release during reperfusion, as the restored blood flow induces shear stress, thereby stimulating endothelial nitric oxide synthase to produce NO. Alternatively, increased endothelium-dependent vasodilation following a single bout of RIC may be associated with other non-NO endothelial mechanisms, such as the induced release of endothelial-derived hyperpolarizing factors (EDHFs) and PGI2 [6,7,8].

Some studies have highlighted the role of autonomic nervous system activation induced by RIC in reducing ischemic reperfusion injury. A previous study found that administering an intravenous infusion of trimethaphan to induce ganglionic blockade eliminated the protective effect of RIC against vascular ischemic reperfusion injury [9]. In contrast, some reports have shown that a single bout of RIC does not alter sympathetic activity [10]. Thus, the effects of RIC on the autonomic nervous system and endothelium-dependent vasodilation may influence the cardiovascular response to specific maneuvers, such as passive leg raising (PLR).

PLR is used as a self-fluid challenge to optimize fluid therapy by predicting the preload responsiveness in critically ill patients with hypotension [11]. In non-critically ill or healthy individuals, PLR can be used to assess arterial vasodilator reserve, endothelium-dependent vascular function, and baroreflex sensitivity [12]. PLR, performed by shifting an individual from a semi-recumbent to a supine position with elevated legs, can induce cardiovascular responses not only through increased cardiac preload due to blood shifting from the legs but also by a sympathetic effect resulting from nociceptive stimulation, baroreflex activation [13], and endothelial response to shear stress [12]. Regarding preload responsiveness, a clinically significant change is defined as an increase in the cardiac output (CO) or stroke volume (SV) by 10–15% during a PLR test [14,15,16].

Meta-analyses of clinical trials and studies in healthy adults have found no significant reduction in diastolic or mean arterial pressure following a single session of RIC [17]. However, not all studies specified the exact timing of their hemodynamic measurements after the RIC procedure. Additionally, studies have demonstrated that a single RIC session can increase flow-mediated dilatation (FMD) or preserve FMD in the face of ischemic reperfusion injury [5,7]. This beneficial effect on endothelial function has been observed during two distinct periods: an early protective window (approximately within 4 h post-RIC) and a delayed protective window (beginning about 24 h and lasting up to approximately 72 h after the RIC) [5,9]. Thus, a single RIC intervention appears to induce two separate phases of enhanced endothelial reactivity. Another study reported that RIC influences the hemodynamic response to metaboreflex activation by attenuating the blood pressure response, primarily due to a reduced SV response [18]. The authors concluded that RIC modulates hemodynamics mainly by impairing the ability to enhance venous return and by limiting the recruitment of the cardiac preload reserve.

Overall, the short-term effects of RIC on the cardiovascular responsiveness to the PLR test are unknown. Therefore, in this study, we aimed to evaluate whether a single bout of RIC alters the PLR-induced cardiovascular response.

## 2. Materials and Methods

### 2.1. Participants

We included 36 healthy, recreationally active volunteers (25 women and 11 men; median age, 24 years; Table 1). Patients with acute illness, known cardiovascular disease, or risk of thromboembolism were excluded from participation. The participants were instructed to abstain from caffeine, alcohol, and tobacco for 12 h before the experiment. To ensure uniformity, all the measurements were conducted between 8:00 and 11:00 a.m.

### 2.2. Experimental Procedure

Hemodynamic parameters were assessed using an ICON monitor (Osypka Medical GmbH, Berlin, Germany), which employs the Electrical Cardiometry method to estimate SV and CO through noninvasive, continuous measurements of thoracic bioimpedance. This approach detects fluctuations in thoracic electrical bioimpedance arising from changes in blood conductivity within the aorta, attributed to the dynamic alignment and reorientation of red blood cells during the cardiac cycle [19]. Four electrodes were placed on the neck and chest according to the manufacturer’s instructions: two on the left side of the neck and two on the left side of the lower thorax. These skin electrodes facilitate continuous thoracic bioimpedance monitoring by transmitting a low-amplitude, high-frequency electrical current through the chest. The SV was calculated on a beat-by-beat basis and averaged over 10 s. The Electrical Cardiometry technique has been validated across various populations, including individuals undergoing exercise [19], selected critically ill patients [20] (compared with thermodilution), preoperative patients [21], and pediatric cohorts [22].

To obtain photoelectric plethysmographic signals for perfusion index (PI) measurement, a reusable pulse oximeter sensor was placed on the third or fourth finger of the hand contralateral to the arm on which the noninvasive blood pressure cuff was placed. The pulse oximeter sensor was connected to an IntelliVue MX100 monitor (Philips Medical Systems, Amsterdam, Netherlands). The PI values were used as surrogate measures for arteriolar vasomotor tone. The PI was measured during PLR before and after RIC.

**Figure 1:**
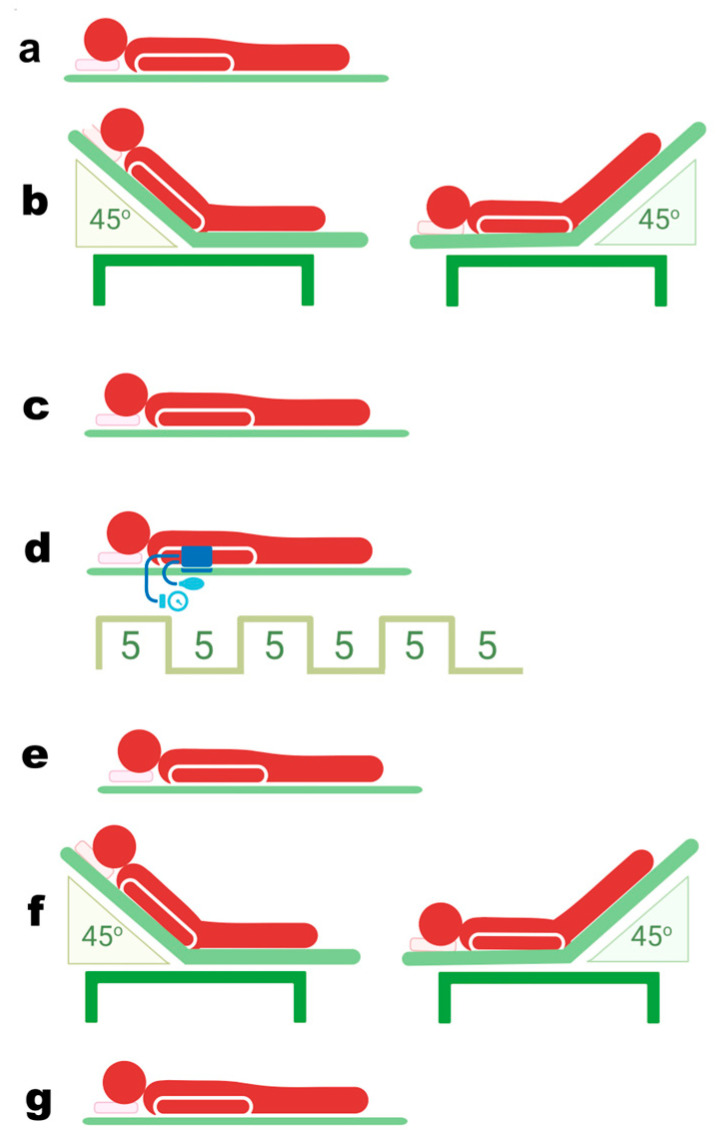
Overview of study procedures: (**a**), supine position; (**b**), first passive leg raising (PLR); (**c**), rest in the supine position; (**d**), remote ischemic conditioning; (**e**), supine position; (**f**), second PLR; (**g**), rest in the supine position. This image was created with BioRender.com.

Systemic hemodynamic indices (heart rate (HR), arterial blood pressure, SV, and CO) were assessed through the following sequential steps (Figure 1): with the participant in a supine position, during the first PLR maneuver (PLR I), before RIC, after RIC, during the second PLR maneuver (PLR II), and with the participant in a supine position.

Baseline parameters were measured after the participant rested for 5 min in a supine position. This was followed by the PLR maneuver. The participants were positioned in a semi-recumbent position for at least 2 min, with the bed’s backrest inclined at a 45° angle. They were then transitioned to a supine position, and both legs were elevated to a 45° angle from the bed for another 2 min. The highest SV measured during the PLR test was recorded. Preload responders and non-responders were defined as those having a SV increase of ≥10% and <10%, respectively. After completing the PLR test, the participants were placed in the supine position for 5 min. Thereafter, RIC was performed. The RIC protocol consisted of three cycles of upper hand compressions using a blood pressure cuff at 200 mmHg for 5 min per cycle, followed by 5 min of reperfusion. The systemic hemodynamic parameters were measured immediately after RIC. The PLR test was repeated, after which the participants were placed in a supine position for 5 min at rest and measurements were taken.

### 2.3. Statistical Analysis

The primary outcomes were the proportion of responders (with SV increase ≥ 10% during PLR) and the correlation between SV changes during PLR before and after RIC.

The sample size was calculated based on the SV data obtained from our observations and results from previous studies involving similar populations [23]. Assuming a mean SV of 90 mL in the semi-recumbent position, we estimated that at least 21 patients were needed to detect a clinically relevant difference of 9 ± 11 mL (10% increase in SV) during the PLR test with a power of 90% and an alpha level of 0.05. Our study had a power of 90% and a two-sided alpha level of 0.05; hence, this study could detect a strong correlation (Spearman’s correlation coefficient ≥ 0.6) between SV changes during PLR before and after RIC using a minimum of 30 participants.

The statistical analyses were conducted using SPSS software (IBM Corp., version 27, Armonk, NY, USA). The normality of the data was assessed with the Shapiro–Wilk test. As most data did not follow a normal distribution, the results are presented as medians with interquartile ranges. Differences in parameters during PLR before and after RIC were assessed using the Wilcoxon signed-rank test. For multiple related datasets, the Friedman test was employed, followed by Bonferroni-corrected pairwise comparisons using the Wilcoxon signed-rank test. The McNemar test was utilized to evaluate differences between two related groups for dichotomous dependent variables, such as preload responsiveness. Correlations were analyzed using Spearman’s correlation coefficient. A *p*-value of less than 0.05 was considered statistically significant.

## 3. Results

### 3.1. Effects of RIC on Systemic Hemodynamics

**Figure 2:**
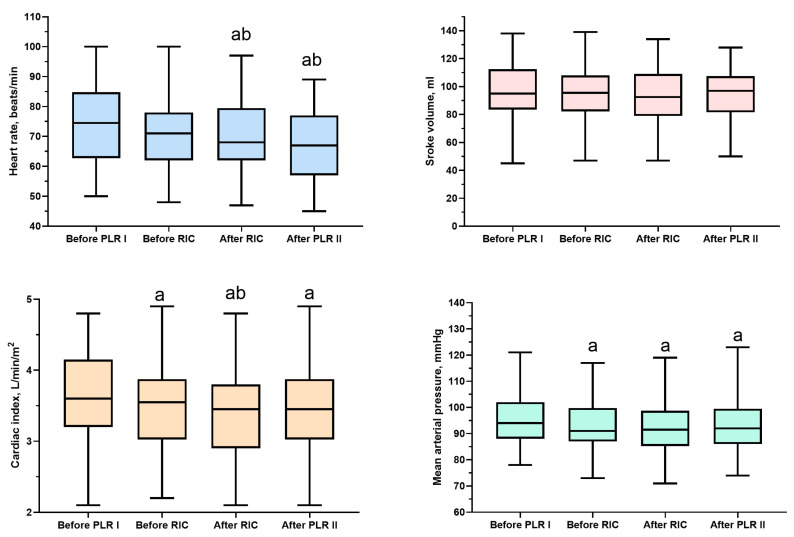
Boxplots of hemodynamic parameters measured during study time points when participants were in the supine position without interventions. ^a^ *p* < 0.001 compared with the value before PLR I; ^b^ *p* < 0.05 in comparison with the value before RIC. PLR, passive leg raising; PLR I, first PLR test; PLR II, second PLR test; RIC, remote ischemic conditioning.

The HR significantly decreased after RIC (HR before vs. after: 71 (62–78) vs. 68 (62–80) beats/min, *p* < 0.001). The reduction in HR persisted even after PLR II (Table 2 and Figure 2). A significant decrease in the cardiac index (CI) was observed following RIC compared with pre-RIC values. RIC had no short-term effects on SV or MAP.

### 3.2. Effects of RIC on PLR-Induced Cardiovascular Response

**Figure 3:**
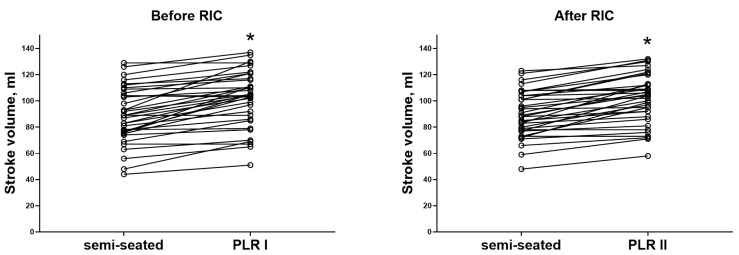
Individual changes in stroke volume during passive leg raising (PLR) before and after remote ischemic conditioning (RIC). * *p* < 0.05.

**Figure 4:**
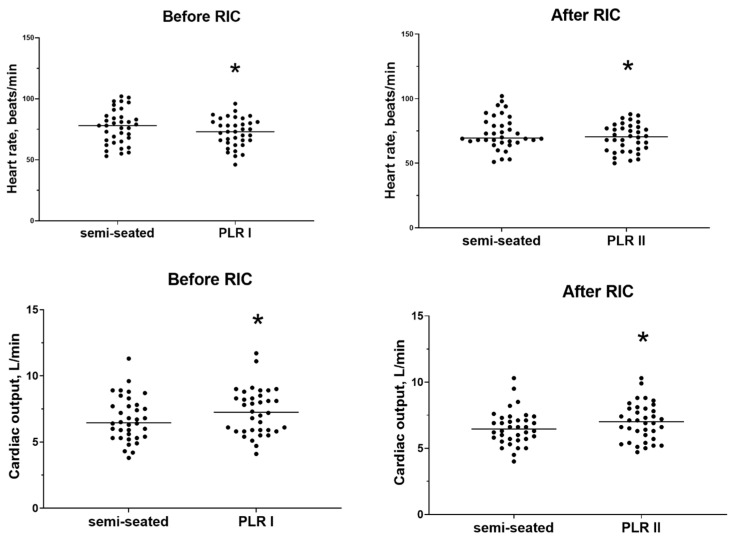
Scatterplots of changes in heart rate and cardiac output during passive leg raising (PLR) before and after remote ischemic conditioning (RIC). * *p* < 0.05.

The participants had a median SV of 89 mL (76–106 mL) in the semi-recumbent position before PLR I, compared with 89 mL (77–104 mL) before PLR II (after RIC) (*p* = 0.325). PLR significantly reduced the HR and increased the SV and CO before and after RIC in a similar manner (Figure 3 and Figure 4). We found no PLR-induced differences in HR (−4 [−9–1] vs. −3 [−9–0] beats/min, *p* = 0.524), SV (11 [4–20] vs. 11 (4–18) mL, *p* = 0.488), and CO (0.4 [0.2–1.1] vs. 0.4 [0.0–1.0] L/min, *p* = 0.838) before and after RIC.

The median SV change was 14% (5–23%) for PLR I and 9% (4–21%) for PLR II. CO changes were 6% (2–15%) and 6% (0–16%) for PLR I and PLR II, respectively. The change in HR followed a similar pattern (−5% [−11–2%] during PLR I and −4% [−12–0%] during PLR II).

A significant negative correlation was observed between changes in HR and SV during PLR I (rs = −0.42, *p* = 0.012), with a trend toward significance during PLR II (rs = −0.30, *p* = 0.08).

**Figure 5:**
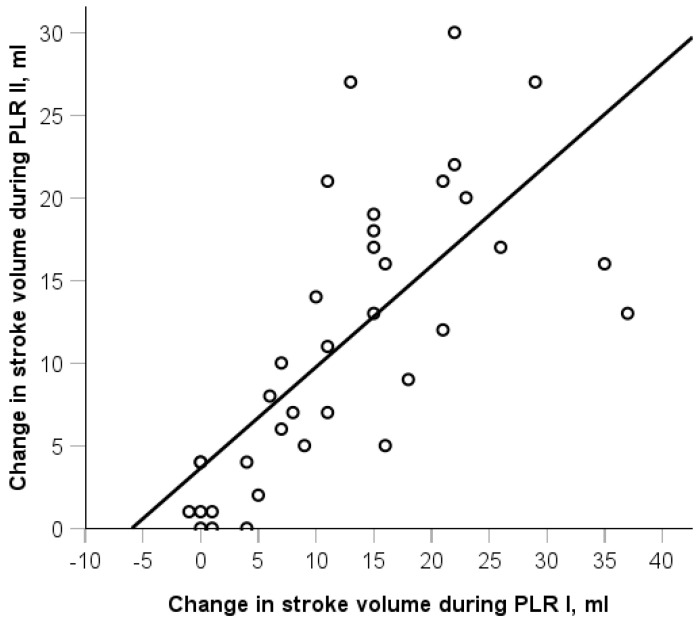
Correlation between the changes in stroke volume during passive leg raising (PLR) before and after remote ischemic conditioning. r_s_ = 0.8, *p* < 0.001.

Additionally, the proportion of responders during the PLR test did not differ before and after RIC (61% vs. 47%, *p* = 0.180). There was a strong correlation between SV changes during the two PLR tests (r_s_ = 0.80, *p* < 0.001, Figure 5).

**Figure 6:**
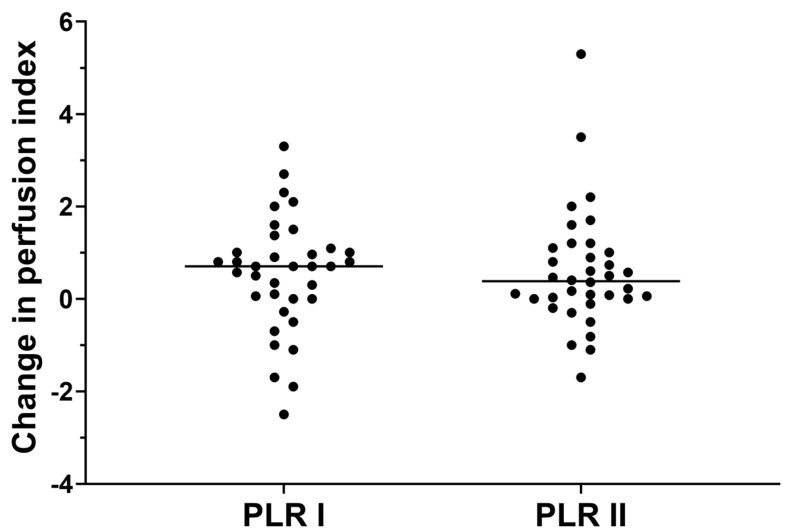
Individual changes in perfusion index during passive leg raising (PLR) before (PLR I) and after (PLR II) remote ischemic conditioning.

Regarding PLR-induced changes in the PI, PLR significantly increased the PI before and after RIC. The PI changes during PLR before and after RIC were 0.7 (0.0–1.1) (*p* = 0.012) and 0.4 (0.0–1.1) (*p* = 0.005), respectively. However, there was no significant difference between the changes in PI during PLR before and after RIC (*p* = 0.944, Figure 6).

## 4. Discussion

To our knowledge, this is the first study demonstrating that a single bout of RIC has no effect on preload responsiveness. We found a similar response during PLR in terms of the proportion of preload responders before and after RIC. In addition, there was a strong correlation between SV changes during PLR before and after RIC. This can be explained by low vasodilator levels (below the required threshold to elicit macrohemodynamic effects) immediately after RIC or short vasodilator half-life in the blood; moreover, it might have been too early for the effect to occur. Considering the very short half-life of nitric oxide, from 0.1 to 5 s, it is likely that a response would occur only in a localized area because there is not enough lifetime to reach remote regions. Studies have demonstrated that repeated bouts of brief vascular occlusion followed by subsequent hyperemia (i.e., forearm occlusion for 5 s and rest for 10 s for a total period of 30 min), which generate episodic shear stress, can enhance endothelial function and NO-dependent vasodilatation in the experimental arm but not in the contralateral (control) arm [24,25]. On the other hand, a study demonstrated that three days of repeated ischemic conditioning (three occlusions lasting 30 s each) restored the brachial artery dilation (BAD) response to PLR, which had been blunted by ischemia–reperfusion injury [26]. In this study, baseline PLR elicited a 3.85% increase in BAD. The changes in BAD in response to PLR were 3.11% under preischemic conditioning and 3.74% under postischemic conditioning. In our study, we used single-bout ischemic conditioning with longer occlusion durations (three occlusions lasting 5 min each, with subsequent reperfusions lasting 5 min); no additional stimulus for ischemic reperfusion injury was applied. We used PLR-induced changes in PI to evaluate endothelial function. During the PLR test, the PI increased by 22.4% and 35.2% before and after RIC, respectively. The PI was calculated as the ratio of the pulsatile component (representing the arterial compartment) to the non-pulsatile component (comprising venous, capillary, and tissue compartments) of the light detected by the pulse oximeter sensor [27,28]. Peripheral vasodilation primarily enhances the pulsatile component, which directly influences the previously mentioned ratio, leading to an increase in the PI.

Studies using RIC protocols with longer periods of ischemia and reperfusion (i.e., 5–10 min occlusion and 5–10 min reperfusion periods) have reported a remote effect of improved cutaneous microcirculation for at least 4 h after RIC [29] and increased vasodilation in response to acetylcholine or local heating after 24–48 h [30]. The remote effect of a single bout of RIC may be associated with increased bioavailability of NO [31] during ischemic periods or other non-NO endothelial factors (such as EDHF and PGI2). EDHF is responsible for protection during the first window, whereas PGI2 is involved in the second window [32].

Most previous studies examined the function of small vessels or microvessels before and after RIC. However, the RIC-induced changes in systemic hemodynamics and circulatory reactivity remain unclear. In our study, RIC decreased the HR and CI, which indicates vasodilation and/or an increase in parasympathetic activity and a decrease in sympathetic activity after RIC. However, an unaltered response to PLR after RIC may partially negate the suppressed sympathetic activity. A previous study demonstrated that using trimethaphan infusion as a ganglionic blockade abolished the protective effect of RIC [9], thereby highlighting the role of the autonomic nervous system in modulating the protective effects of RIC. Lambert et al. found that RIC attenuates ischemia-induced sympathetic activation, prevents the production of an erythrocyte marker of oxidative stress, and reduces NO availability [33]. However, studies have reported opposing effects of a single bout of RIC on sympathetic alterations [10,18]. Another study reported an increase in SD2, a measure of heart rate variability (HRV), and suggested that both the parasympathetic and sympathetic nervous systems were activated—through a rapid vagal (parasympathetic) response and a slower sympathetic response triggered by baroreceptor stimulation [34]. Regarding the delayed or late window of protection, a single bout of RIC may be insufficient to induce subsequent sympathovagal effects. Gardner et al. indicated that repeated RIC (for 2 weeks) is necessary to elicit changes in the sympathovagal balance; this increases vagal activity and decreases sympathetic activity [35].

We found no changes in the mean arterial pressure (MAP) before and after RIC. This finding corroborated that of a previous meta-analysis that reported that repeated, not single, RIC produced clinically meaningful reductions in MAP [17]. However, the possible effect of PLR on MAP should also be considered because PLR neutralizes the influence of RIC. Some studies have shown that PLR may lower blood pressure in healthy participants by increasing preload and activating low-pressure cardiopulmonary baroreceptors. This results in smooth-muscle-mediated arterial dilation and higher CO-induced brachial artery shear stress, as well as brachial artery endothelial-dependent vasodilation [12,36]. Although some studies had contrary findings, it is difficult to compare these findings with our findings because we measured the MAP with the participants in the supine position.

PLR is a simple diagnostic test used to assess baroreceptor function, detect subclinical left ventricular dysfunction, measure arterial vasodilator reserves and endothelial function, and measure baroreceptor sensitivity [12]. Leg raising is used to evaluate fluid responsiveness and temporarily increase blood pressure in critically ill patients with hypotension [37]. During PLR, intrathoracic blood pooling reduces sympathetic activity and enhances parasympathetic activity, leading to a decrease in HR [38]. This finding is consistent with our study findings; during PLR, the HR decreased significantly.

Our study has some limitations. We did not include a sham group, as it is not feasible to design an intervention that closely replicates the RIC maneuvers. Some previous studies have attempted this by inflating a blood pressure cuff to pressures between 20 and 60 mmHg [39,40,41]. However, the participants can clearly perceive the inflation of the cuff when it is inflated to higher pressures, specifically between 20 and 50 mmHg above systolic pressure [18]. Furthermore, even mild cuff inflation could potentially reduce venous return and cause metabolite accumulation, paradoxically introducing additional confounding factors. On the other hand, the minimal impact of time on the validity of our protocol is supported by a previous study involving healthy volunteers that demonstrated a strong correlation in SV changes between two consecutive PLR maneuvers [23]. Moreover, we neither measured NO production nor recorded the HRV. Nevertheless, we measured the PI as an indicator of vasomotor tone, and its change during PLR indicated endothelial-dependent vasodilation. Additional markers of endothelial function, such as FMD, would strengthen the findings derived from the PI changes. FMD is the noninvasive gold standard for assessing endothelial function and is a well-established predictor of cardiovascular events [42]. Typically, FMD is measured using high-resolution duplex ultrasound at the brachial artery, where vasodilation following a 5 min suprasystolic occlusion and subsequent reactive hyperemia reflects a shear-stress-induced, nitric oxide-mediated response. Prior research has shown that PLR-induced brachial artery dilation is significantly correlated with FMD, although its magnitude is less than half that observed with FMD [12]. Therefore, PLR-induced brachial artery dilation may serve as a surrogate measure of FMD for evaluating endothelial function. Additionally, another study demonstrated that, following reactive hyperemia, the data derived from the oximetry plethysmographic curve displayed patterns similar to those obtained using brachial artery Doppler assessments [43].

Electrical Cardiometry has demonstrated validity during exercise when compared with the Fick method [19]. However, its clinical utility in critically ill and high-risk surgical patients remains a subject of debate. Electrical Cardiometry has shown a trend toward improved performance in patients with less severe critical illness, as reflected by a lower percentage error and a higher concordance rate in tracking CO during transpulmonary thermodilution [44]. Nevertheless, a recent study reported that, in patients undergoing coronary artery bypass graft surgery, the agreement between CO measurements obtained via Electrical Cardiometry and those obtained using intermittent pulmonary artery thermodilution was not clinically acceptable [45].

Without a direct evaluation of sympathetic activity, it is difficult to draw any conclusions about the PLR-induced reflex changes after RIC. Finally, the study population was young and healthy; therefore, the results cannot be generalized to patients and elderly people.

## 5. Conclusions

Our study suggests that a single bout of RIC has no effect on the cardiovascular response to PLR in terms of changes in systemic hemodynamic and peripheral perfusion indices. RIC has some effects on systemic hemodynamics, such as a decrease in HR and CI.

## Figures and Tables

**Table 1:** Baseline participant characteristics.

	Value
Sex, female, n (%)	25 (69)
Age, years	24 (23–26)
Body mass index, kg/m^2^	24.0 (21.6–27.2)
Heart rate, beats/min	75 (63–85)
Mean blood pressure	94 (88–103)
Cardiac index, L/min/m^2^	3.7 (3.2–4.2)
Stroke volume, mL	96 (84–113)

**Table 2:** Hemodynamic parameters measured during study time points when participants were in the supine position at rest.

	Before PLR I	Before RIC	After RIC	After PLR II	*p*
Heart rate, beats/min	75 (63–85)	71 (62–78)	68 (62–80) ^ab^	67 (57–77) ^ab^	0.002
Mean blood pressure, mmHg	94 (88–103)	91 (87–100) ^a^	92 (85–99) ^a^	92 (86–100) ^a^	0.049
Cardiac index, L/min/m^2^	3.7 (3.2–4.2)	3.6 (3.0–3.9) ^a^	3.5 (2.9–3.8) ^ab^	3.5 (3.0–3.9) ^a^	0.001
Stroke volume, mL	96 (84–113)	96 (82–108)	93 (79–109)	97 (82–108)	0.445

^a^ *p* < 0.001 compared with the value before PLR I; ^b^ *p* < 0.05 in comparison with the value before RIC. PLR, passive leg raising; PLR I, first PLR test; PLR II, second PLR test; RIC, remote ischemic conditioning.

## Data Availability

The datasets generated in this study are available from the corresponding author upon request.

## Abbreviations

The following abbreviations are used in this manuscript:

RIC	Remote ischemic conditioning
NO	Nitric oxide
FMD	Flow-mediated dilatation
PLR	Passive leg raising
CO	Cardiac output
SV	Stroke volume
CI	Cardiac index
HR	Heart rate
MAP	Mean arterial pressure
HRV	Heart rate variability
BAD	Brachial artery dilation
